# The Relationship between Mental Health, Educational Burnout and Strategies for Coping with Stress among Students: A Cross-Sectional Study of Poland

**DOI:** 10.3390/ijerph182010827

**Published:** 2021-10-15

**Authors:** Piotr Długosz, Damian Liszka

**Affiliations:** Institute of Philosophy and Sociology, Pedagogical University of Krakow, 30-084 Krakow, Poland; damian.liszka@up.krakow.pl

**Keywords:** COVID-19, psychosomatic disorders, educational burnout, philosophy of education challenges, coping, students, Poland

## Abstract

This study sought to investigate the risk factors of poor psychosomatic health among students during the quarantine of the first wave of the COVID-19 pandemic. A survey was conducted on-line, on a sample of 1978 respondents in Poland. The study was carried out towards the end of the summer 2020 semester. The questionnaire used in the study was designed so that it allows for the observation of the main risk factors which have an impact on the students’ mental health. Variance analysis and hierarchical regression analysis were used to determine the predictors of mental health problems. The results indicate that average and high levels of psychosomatic disorders were observed among 61% of respondents. The hierarchical regression analysis showed that an increase in the level of educational burnout, a decreased life satisfaction, and use of negative strategies of coping with stress, were accompanied by a deteriorated mental condition of students. Moreover, it was observed that female respondents scored higher on the scale of disorders in comparison to males.

## 1. Introduction

The coronavirus pandemic has been described as a perfect storm with a tremendous aftershock of psychosocial and economic effects that cannot be fully discovered and predicted. At the moment, a decrease in the mental and physical health indices and an increase in economic inequalities are expected [1]. The imposition of a lockdown has led to the closing of schools and universities all over the world. It is estimated that approximately 1.5 billion learners—representing 91% of students in the world—have suffered as a result of the imposition of distance education [2]. Turning to distance learning on a global scale leads to the risk of an increase in educational inequalities and a deterioration of the students’ mental health. It is worth bearing in mind that not only do schools have an educational function, but they also provide nutrition and health services, especially those related to mental health. It is estimated that as a result of the closing of schools in the USA, approximately 3 million teenagers got deprived of psychological assistance [3]. This may be the reason for the significant deterioration of the mental health of young people in many countries [4,5,6].

Studies conducted in many countries indicate that during the quarantine students displayed high levels of stress, anxiety and depression [7,8]. Different studies have also shown that apart from the symptoms of distress, students declared suicidal thoughts, problems with concentration or self-mutilation [9]. In the USA, 71% of students maintain that during the pandemic they observed an increase in their levels of stress and anxiety [7]. Meta-analyses indicate that 9–54% of students all over the world experienced psychological distress [8]. 

Analyses have shown that the main mental health risk factors during the pandemic are: being a female [9], living on one’s own [9,10], an uncertain financial situation [9], contact with people who contracted COVID-19 [8,9], being in the final year of studies [8,11], lack of social support [9,12], social media exposure [9] and using negative strategies for coping with stress [13]. What is more, studies indicated that being overloaded with distance education has a negative impact on the mental health of youth [11].

The vast majority of studies are of cross-sectional nature and therefore it is impossible to unambiguously determine that the pandemic and its consequences have led to a deterioration of the mental health of young people. Nonetheless, there are results of comparative research that indicate that the mental health of youth has deteriorated during the pandemic [4,5,6]. There are also studies carried out in Poland with results that confirm a negative impact of the lockdown on young people’s mental health. In the age group 18–24, 26% of respondents experienced depression and being unhappy in 2019, whereas 32% of respondents felt depressed and unhappy in 2020. The feeling of helplessness and fatigue was experienced by 15% of respondents in 2019 and 44% of respondents in 2020. The feeling of fatigue and lack of motivation was declared by 27% of respondents in 2019 and 47% of respondents in 2020 [14]. 

The majority of research conducted thus far on the mental health of youth was carried out among secondary school students [15,16,17]. This study sought to delve into the situation of students whose lives changed dramatically during the quarantine. They returned to their homes, got deprived of contact with their teachers and friends, and spend long hours in front of their computer screens. Their social lives have been limited significantly as well. Additionally, socio-economic circumstances may have an impact on the mental health of students. One should bear in mind that COVID-19 is a dangerous disease and the fear of it may have a negative impact on the students’ health. 

The pandemic posed a real threat to one’s life and health. While this study was being conducted in June 2020, there were approximately 300 confirmed COVID-19 cases a day. This increased to 700 new cases a day in August, and more than 800 cases a day in September. The second wave started in October 2020 and the number of reported incidences increased to 9000 cases a day [18]. In Poland and in other countries, there has been an increase in deaths as a result of COVID-19. The COVID-19 mortality rate was 2.2% [19]. In June 2020, around 820 people per million inhabitants had been diagnosed as infected, as compared with the following numbers of cases per million in other countries: USA, 6900; Belgium, 5200; UK, 4400; Italy, 3900; but only 280 in neighboring Slovakia [20].

Moreover, the economic context is important, as the lockdown might have led to an economic crisis and a decrease in the sense of economic security. This is evident in the research showing that in Poland young people suffered the most during the lockdown. Research shows that while 9% of Poles experienced a reduction in employment and had to limit their economic activity, 11% of young people aged 18–24 were forced to limit their economic activity and suffered a reduction in employment. Moreover, 3% of Poles lost their jobs during the lockdown, and 10% among young people. The situation was similar with reduced hours at work. Among the total number of respondents, working time limitations affected 20% of Poles, and among young people, it was 28% [21]. It was even more difficult for youth in the United States, wherein the age group of 16–24 the unemployment rate has increased from 8% in January 2020 to 25.3% in April 2020 [22].

Moving on to the conducted research, it is worth emphasizing that the aim of the research was to check the psychosomatic condition of students who had participated in distance education for several months. The study was meant to answer the question of whether the pandemic and distance education have a negative impact on the mental health of adolescents.

It is assumed that the main factor negatively influencing the mental health of students is being exhausted from distance education. The higher the level of educational burnout, the worse the psychosocial condition of students. The strategy for coping with stress may also determine the students’ psychological condition. In the case of using emotion-focused strategies, a deterioration of mental health may occur. Socio-demographic features influence one’s psychosomatic condition to a lesser extent. 

## 2. Methods

### 2.1. Sample

This cross-sectional study was conducted using a Computer-Assisted Web Interview to assess mental health problems. The research survey was conducted on-line between the 1 June 2020 and the 10 June 2020. The questionnaire was placed on the LimeSurvey platform. The study was conducted by the end of the summer semester on purpose, in order to determine the impact of distance education on the students’ mental health in more depth. The survey was conducted on a sample of 1987 students of the Pedagogical University of Kraków. In Kraków, there are 21 universities, in which more than 150,000 people were studying in the 2019/2020 academic year, of which the Pedagogical University of Kraków numbered around 10,000 students. 

A non-random sampling method was used in the study. Convenience sampling was based on the availability of surveyed students. The respondents were selected by means of sending a link to the survey. The study was voluntary and anonymous. All procedures were conducted in accordance with the ethical standards of the Helsinki Declaration of 1975, as revised in 2008. 

The questionnaire included questions regarding the following demographic factors: gender, personal evaluation of one’s financial standing, place of birth and place of residence during the time of this research study, university major, year of studies, social status, religiosity and university grades. In the study, attitudes towards the pandemic, satisfaction with life and strategies for coping with stress were measured as well. 

### 2.2. Measured Variables

#### 2.2.1. The Scale for Measurement of Psychosomatic Disorders (HBSC-SCL)

The scale used to measure health disorders was derived from the HBSC-SCL research [23], which was modified for the purpose of the Polish HBSC research [24] and adapted to the research at hand. The following eight symptoms were measured: headache, stomach ache, dizziness, trouble sleeping, nervousness, gloom, bad mood, fatigue and irritability. The intensification of symptoms in the last 7 days was evaluated using an ordinal scale, which was developed by the authors of the research. The responses are as follows: yes, several times—4; yes, a few times—3; yes, 1–2 times—2; I haven’t felt—1. The Cronbach reliability score was *α* = 0.864.

#### 2.2.2. Educational Burnout

Educational burnout was defined as exhaustion due to stress and pressure which stem from the tasks and duties fulfilled by the students in their school and educational classes [25] This research used the LBQ Link Burnout Questionnaire [26] in the Polish version [27]. The questionnaire was adapted to the needs of the present research on educational burnout during distant learning. The scale used for measuring educational burnout had 16 items. The measurement was based on a five-point Likert scale, ranging from “I strongly agree” to “I strongly disagree.” The Cronbach reliability score was α = 0.890. 

The respondents were also asked for the evaluation of distant learning conducted within their major. The scale consists of the following answers: 1—very good, 2—good, 3—average, 4—bad, 5—very bad. 

#### 2.2.3. Strategies for Coping with Stress 

The construct put forward by Lazarus and Folkman [28] was used to measure the strategies for coping with stress. Its Polish version was used in the research conducted among students [29]. Dominant strategies were distinguished by means of factorial analysis. The problem-focused stress coping strategy included the following responses: (1) I ask other people for help and advice; (2) I get mobilized and do my best to protect myself from stress; (3) I comfort myself with a thought that it could have been worse, but at the moment I am healthy; (4) I pray to God for help; (5) I focus on different things which divert my attention and improve my mood. The emotion-focused strategy was indicated by the following responses: (1) I use alcohol, drugs, other psychoactive substances; (2) I give up, don’t know what to do and what to expect; (3) I take sedatives.

#### 2.2.4. Financial Stress 

Financial stress was measured by means of a question of whether the COVID-19 pandemic led to a deterioration of one’s financial standing: definitely yes—1, yes—2, no—3, definitely not—4, hard to say—5. 

#### 2.2.5. Satisfaction with Life

Satisfaction with life was measured using the following ordinal scale: very satisfied—1, rather satisfied—2, rather dissatisfied—3, very dissatisfied—4, and hard to say—5. 

#### 2.2.6. Attitudes towards the Pandemic 

The level of interest in the pandemic was measured using the following ordinal scale: highly interested—1, rather interested—2, rather not interested—3, and not interested at all—4. 

The respondents were also asked whether the pandemic poses a serious threat for them: 1—yes, it poses a serious threat, 2—it poses a minor threat, 3—it does not pose a threat, 4—hard to say. 

The attitude towards the government was measured as well. The respondents were asked whether the government and relevant services undertook satisfactory measures in order to protect the country from the coronavirus epidemic or not: 1— yes, they do enough, 2— no, they don’t do enough, 3—hard to say. 

All statistical analyses were conducted using SPSS Statistics version 25. Firstly, we conducted a descriptive statistical analysis of the scale of psychosomatic disorders. Secondly, we assessed associations between demographic data and other variables, which were measured with the HBSC-SCL scale by using the analysis of average values. Finally, a hierarchical regression analysis was used in order to identify the most relevant risk factors determined in the study, taking into account the social and economic contexts. 

## 3. Results

On the applied HBSC-SCL scale of psychosomatic disorders, the result of 18.3 was obtained (SD = 5.6), median = 18, and dominant = 17. The minimum value on the scale was 8, and the maximum value was 32. Considering the obtained results, the students may be divided into three categories: those who had low levels of psychosomatic disorders—score: 8–16 (39%), medium levels—score: 17–24 (46%), and high levels—score: 25–32 (15%). 

The results of the conducted analyses indicate that ¾ of students had health problems and experienced internalizing disorders by the end of distance education. 

Table 1 presents how independent variables influence the value of average results on the HBSC-SCL scale. Female students, those who evaluate their financial status as poor, the students of first-cycle and long-cycle master’s degree studies, those with lower social status and non-believers as well as undecided in terms of faith had worse psychosomatic health. The most significant differences were observed in the case of educational burnout. A high level of educational burnout; bad evaluation of distance education; passive, emotion-focused adaptive strategy, financial stress, lack of satisfaction with life, fear of contracting coronavirus and negative evaluation of activities undertaken by the government intensified the risk of a bad psychosomatic condition. 

Table 2 presents the results of the hierarchical regression analysis which was used in order to investigate the relationship between socio-demographic variables, attitudes towards the pandemic, psychological variables, other variables describing the experience with distance education and the level of psychosomatic disorders. In the first stage, the socio-demographic variables were introduced, in the second stage, the variables measuring the attitudes towards the pandemic were applied, in the third stage, the psychological variables were introduced to the model, whereas in the fourth stage, the variables monitoring the educational burnout were included. 

The results of the hierarchical regression analysis indicate that demographic variables explain only 4% of the psychological discomfort variance. Females, people who evaluate their financial standing as bad, and non-believers experienced higher levels of stress. 

The introduction of variables measuring the attitudes towards the pandemic failed to increase the level of explained variance. In the third stage, upon the introduction of psychological variables, the level of explained variance was 21%. The change in the explained variance was statistically relevant. A significant change in corrected R^2^, (delta corrected R^2^ = 0.167; F change (4,1517) = 80.923, *p* < 0.000) was observed. In this model, the strongest predictor of psychosomatic disorders was the lack of satisfaction with life and an emotion-focused strategy for coping with stress. From the first model, only the gender remained statistically relevant. 

In the fourth stage, upon the introduction of variables describing exhaustion with distance education, the explained variance factor was 41%. The change in the explained variance was statistically relevant. A significant change in corrected R^2^, (delta corrected R^2^ = 0.199; F change (2,1515) = 259.462, *p* < 0.000) was observed. In this model, the strongest predictor of health disorders was exhaustion from distance education. From the first model, only the gender remained statistically relevant. From the third model, the lack of satisfaction with life and the emotion-focused strategy were statistically relevant. 

## 4. Discussion 

The results of this research indicate that psychosomatic health problems are present among Polish students. Internalizing disorders occur as well. After four months of quarantine, almost ¾ of respondents displayed average and high levels of symptoms of internalizing disorders. In a similar study carried out among students in Wrocław, 77% of the participants had psychopathological symptoms measured by the GHQ (28) [30]. This means that the quarantine, the lack of contact with peers and teachers, the stress occurring in their families and uncertainty imposed by the COVID-19 pandemic have a negative impact on the mental health of students in different cultures. This is supported by the results of the meta-analysis in which 706,415 students from all over the world participated. As a result, it was found that the pandemic negatively influenced the mental health of adolescents suffering from depression in 39% of participants (95% CI: 27–51%) and anxiety in 36% of participants (95% CI: 26–46%) [31]. It should also be assumed that the pandemic could activate mechanisms leading to mental health disorders in people without mental health problems and aggravate the problem of young people who already had problems with mental health. According to WHO statistics, the global prevalence of depression in 2015 was 4.4%, and the prevalence of anxiety was 3.6% [32]. On the other hand, the results of comparative studies in the United States show that before the pandemic, among people aged 19–29 between 2018 and 2020, the percentage of moderate or serious distress increased from 26% to 82% [4]. The analyses conducted within this study also allow for the identification of the main sources of psychological distress among students during the lockdown. Educational burnout had the strongest impact on internalizing disorders. Social isolation, lower motivation, the lack of contact with peers and teachers, exhaustion from learning on the computer, and the lack of physical activity constitute the educational burnout syndrome. The above findings are observed in other studies [33,34]. A differing study, indicating a decrease in educational burnout among students during the quarantine, has also come to being [35]. 

Yet another risk factor of a bad psychosocial condition was the passive, emotion-focused strategy for coping with the pandemic-related stress [28]. The students who undertook strategies such as using psychoactive substances, sedatives, and passively observing events in the world have worse psychosomatic health. The confirmation of the above findings can be found in the works of other researchers [13]. A strong relationship between negative adaptive stress coping strategies and higher levels of anxiety, stress and depression is discussed therein. Negative strategies may result in the strengthening of the feeling of losing control over one’s life and increase the sense of helplessness. In a situation such as a pandemic, such attitudes will have a negative impact on mental health. 

Satisfaction with life had a positive impact on the students’ mental health, which was indicated in other studies as well [36,37]. It may be assumed that psychological well-being is a deeper personality trait and constitutes protection from pandemic-related stress. 

The results of this research confirm prior assumptions that young females are more likely to display mental disorders during the pandemic. This is also visible in longitudinal studies conducted during the pandemic in Poland. Over the course of five measurements, the levels of depression, anxiety and stress were significantly higher among female students [38]. Similar results were obtained in international studies of students, where statistically significantly higher scores on the Generalized Anxiety Disorder (GAD-7), Patient Health Questionnaire (PHQ-8), and Perceived Stress Scale (PSS-10) were observed in females students [39]. In the United States, female students were significantly more likely to report anxiety and PTSD risk symptoms [40].

The results of this research failed to confirm the relationship between the year of studies, the type of studies/major and an increase in psychosomatic disorders. It is in contradiction to the results of the meta-analysis [41]. Researchers also point out that the risk factors for mental disorders among students include the process of switching from education to the labor market. Changing a known environment and social role is stressful under normal conditions [42]. It would seem that the pandemic increased the scope of risk and uncertainty in the labor market, primarily among young people, because, as research shows, they lost their jobs more often and had a greater problem with employment during the pandemic [22].

Senior students have a similar level of health disorders as students who have just taken up their studies. Furthermore, the results of this research indicate that students returning to their family homes and living with their parents did not result in mental discomfort. This may mean that living with their families did not result in the occurrence of domestic violence, which is also indicated by a range of studies [43,44]. The conducted study indicates that loneliness occurring during distance education has a much stronger impact on young people’s mental health. This is confirmed by the results of the meta-analysis carried out during the pandemic. Importantly, mental health disorders were more closely correlated with the duration of loneliness than with its subjective strength [45]. This means that subsequent lockdowns may aggravate the emerging mental health problem of adolescents.

## 5. Conclusions

This study, conducted among students in a large academic city, indicates that more than half of the respondents report psychosomatic symptoms. Educational burnout was found to be the main risk factor of poor mental health. Therefore, it shall be assumed that being overloaded with distance education is one of the threats to the students’ mental health. This could be observed by the end of the semester during the first wave of the pandemic in Poland. 

In connection with the emerging psychosomatic ailments, it should be assumed that attention, concentration, thought processes and memory will be negatively affected. This will translate into a decline in learning results. Thus, students may become aware of the decline in their educational and life chances. Consequently, this will lead to further negative psychosocial effects that will hinder adaptation to the conditions created by pandemics and present in the post-pandemic world. It is worth adding that this is a particularly dangerous situation for the society, because students are a factor which revitalizes society and are the main force of its development. The main message from this research is also the need to provide psychological support at universities and in the students’ place of residence. The results of this research clearly indicate that the authorities of countries, local governments and universities should immediately start providing psychological help for young people. The knowledge, obtained from many studies, that indicates how to improve the mental condition of students and how to minimize risk factors should be used. The university authorities should enable the use of online psychotherapy. University authorities should also strive to improve their well-being and teach students effective strategies for coping with stress.

The scale of problems caused by remote education and the pandemic also shows the need for in-depth quantitative and qualitative research on young people. Through systematic observation and triangulation of methods, it is possible to determine the psychosocial effects of the lockdown and distance education and to check, for example, through a case study, what are the effective ways of helping students with mental disorders. Evidence-based knowledge would create a catalog of good practices and improve student interventions. 

## 6. Limitations

The present study has certain limitations. Since the data were collected from participants on a voluntary basis through an on-line application, generalizations should be made cautiously. It was also difficult to determine the rate of return of completed interviews due to a link with the survey being sent to the respondents. Another limitation is the cross-sectional nature of this study. The implemented research plan prevents us from drawing causal conclusions regarding the relationship between the impact of the pandemic on the variables measured in the respondents. Finally, it is worth adding that one of the authors is an academic teacher at the university where the research was conducted, and this could somehow affect the answers obtained from the students.

## Figures and Tables

**Table 1:** Relationship between independent variables and mental health.

		n	M	SD	
Gender	Male	321	16.2	5.8	F value = 60.3 *p* value = 0.000 η^2^ = 0.031
	Female	1593	18.8	5.4	
Financial standing	Bad	323	20.3	5.4	F value = 24.3 *p* value = 0.000 η^2^ = 0.025
	Average	1279	18.0	5.5	
	Good	307	17.7	5.7	
Place of residence	Village	816	18.1	5.5	F value = 1.18 *p* value = 0.314 η^2^ = 0.002
	City up to 100,000 inhabitants	442	18.6	5.4	
	City between 100,000 and 500,000 inhabitants	133	18.8	5.1	
	City larger than 500,000 inhabitants	519	18.5	5.8	
Place of residence at the time of the survey	Family home	1465	18.4	5.6	F value = 0.18 *p* value = 0.831 η^2^ = 0.000
	Rented apartment	427	18.5	5.6	
	Dormitory	19	17.7	6.6	
Type of studies	First-cycle studies	1076	18.6	5.6	F value = 5.18 *p* value = 0.006 η^2^ = 0.006
	Second-cycle studies	458	17.7	5.5	
	Long-cycle master’s degree studies	316	18.7	5.5	
Year of studies	First	827	18.5	5.6	F value = 1.13 *p* value = 0.336 η^2^ = 0.002
	Second	511	18.2	5.4	
	Third	290	18.8	5.8	
	Fourth	111	18.4	5.7	
	Fifth	145	17.7	5.3	
Social status	Low	396	19.1	5.4	F value = 6.23 *p* value = 0.002 η^2^ = 0.007
	Medium	1059	18.3	5.6	
	High	385	17.7	5.7	
Faith	Believers	1260	18.1	5.4	F value = 5.45 *p* value = 0.004 η^2^ = 0.006
	Non-believers	322	18.8	5.8	
	Undecided	329	19.1	5.7	
School grades	Up to 3,5	200	19.1	5.6	F value = 2.30 *p* value = 0.100 η^2^ = 0.002
	From 3.51 to 4.5	1474	18.3	5.5	
	Higher than 4.51	211	18.7	6.0	
Educational burnout	Low (≤36)	227	13.2	4.6	F value = 318.06 *p* value = 0.000 η^2^ = 0.254
	Medium (37–58)	1112	17.6	4.9	
	High (≥59)	531	22.3	4.7	
Evaluation of distance learning	Bad	786	16.9	5.6	F value = 46.91 *p* value = 0.000 η^2^ = 0.047
	Average	679	19.1	5.3	
	Good	461	19.6	5.1	
Problem-focused strategy	Yes	783	18.1	5.8	F value = 2.58 *p* value = 0.133 η^2^ = 0.001
	No	1144	18.5	5.4	
Emotion-focused strategy	Yes	1528	21.2	5.8	F value = 136.00 *p* value = 0.000 η^2^ = 0.066
	No	399	17.6	5.3	
Financial stress	Yes	895	19.3	5.6	F value = 28.35 *p* value = 0.000 η^2^ = 0.029
	No	793	17.3	5.4	
	Don’t know	224	18.5	5.3	
Satisfaction with life	Yes	1428	17.3	5.3	F value = 118.52 *p* value = 0.000 η^2^ = 0.111
	No	299	21.9	5.2	
	Don’t know	184	21.1	5.0	
Interest in the pandemic	Yes	1458	18.4	5.6	F value = 0.42 *p* value = 0.516 η^2^ = 0.000
	No	455	18.2	5.4	
Fear of COVID-19	Yes, it poses a threat	405	18.4	5.7	F value = 5.11 *p* value = 0.002 η^2^ = 0.008
	Poses only a slight threat	868	18.6	5.4	
	Does not pose a threat	364	17.4	5.8	
	Hard to say	276	18.9	5.4	
Evaluation of government	Positive	530	17.7	5.3	F value = 8.18 *p* value = 0.000 η^2^ = 0.010
	Negative	534	19.1	5.8	
	Hard to say	696	18.4	5.4	

**Table 2:** Results of multiple regression analyses predicting the level of psychological distress.

	Model I		Model II		Model III		Model IV	
Variables	β	*p*	β	*p*	β	*p*	β	*p*
Gender	**0.150**	**0.000**	**0.142**	**0.000**	**0.123**	**0.000**	**0.092**	**0.000**
Place of residence	0.052	0.072	0.047	0.105	0.026	0.316	0.017	0.464
Place of residence at the time of the survey	0.018	0.530	0.016	0.640	**0.018**	**0.007**	0.007	0.759
Faith	**0.077**	**0.003**	**0.077**	**0.003**	0.022	0.374	0.019	0.369
University grades	0.032	0.210	0.032	0.203	0.006	0.794	0.018	0.365
Relative evaluation of one’s financial standing	**0.118**	**0.000**	**0.113**	**0.000**	0.047	0.080	0.036	0.119
Type of studies	0.036	0.158	0.038	0.134	0.017	0.470	0.001	0.816
Year of studies	0.025	0.340	0.023	0.375	0.015	0.523	0.019	0.342
Social status	0.028	0.338	0.030	0.303	0.036	0.178	0.028	0.222
Interest in the information about COVID-19			0.022	0.394	0.012	0.625	0.013	0.455
Fear of the disease			0.019	0.473	0.037	0.120	0.013	0.531
Evaluation of government			0.025	0.327	0.011	0.648	0.022	0.208
Probability of becoming infected			**0.060**	**0.022**	0.039	0.099	**0.043**	**0.047**
Satisfaction with life					**0.267**	**0.000**	**0.155**	**0.000**
Financial stress					**0.067**	**0.005**	0.028	0.174
Problem-focused strategy					**−0.071**	**0.004**	**−0.073**	**0.001**
Emotion-focused strategy					**−0.266**	**0.000**	**−0.151**	**0.000**
Educational burnout							**0.505**	**0.000**
Evaluation of distance learning							0.038	0.087
F (*p* ≤ 0.000)	8.32		6.36		24.92		57.21	
R square	0.041		0.043		0.210		0.410	
Standard error	05.4		5.4		4.9		4.2	

## Data Availability

Data available on request from the corresponding author.
